# Annotations

**Published:** 1901-07-27

**Authors:** 


### ANNOTATIONS.

### The Open-air Treatment of Fevers.

It seems not impossible that the knowledge which has recently been gained concerning the "open-air" treatment of phthisis may lead to a considerable alteration in the accepted methods of dealing with other febrile diseases. The wonderful effects which are sometimes seen to follow the placing of febrile consumptives in the open and leaving them exposed to all the vicissitudes of an English winter, coupled with the results which have sometimes followed very rough and ready methods in other fevers, all go to show that brick walls, solid structures, and protection from the weather are not matters of that immense importance which they are officially believed to be. For a long time back the Local Government Board acting as the supreme sanitary authority has set its face against temporary buildings for isolation hospitals. And it has been right in so doing. When the temporary structures employed are mere copies in wood or in corrugated iron of permanent hospitals, it is sufficiently obvious that without possessing the advantages of permanent buildings they have all their disadvantages, together with a considerable number of evils peculiar to themselves, while when tents are so constructed as to give the protection which has been supposed to be necessary their proper ventilation becomes an even more difficult problem than is the case with ordinary buildings. But if we are to apply to fevers the principles which are now so generally accepted in regard to the treatment of tuberculosis, much of the difficulty vanishes. If instead of the very elaborate structures which the word "hospital" has of late years come to connote a much more primitive style of shelter is all that is required during the acute stages of the various fevers, it will evidently be a much simpler affair than it has hitherto been thought to provide villages and small towns with such emergency hospitals as may be required to meet epidemic outbreaks of disease. Such temporary shelters will never be ideal and will never be equal to properly constructed hospitals. But all experience goes to suggest that the use of such structures as are here suggested would be far better than the alternatives, which in too many cases are either removal to distant hospitals, a proceeding always full of danger, or home treatment in the cottage, than which nothing can be worse for the patient or more likely to spread the disease throughout the community. The establishment of central depôts from which portable sheds or tents with a trained staff could be despatched, almost at a moment's notice, might enable the authorities to nip in the bud many an outbreak of infectious disease and to save an infinity of suffering.

### The Decadence of Races.

The student of history is apt, correctly or not, to draw certain broad morals from what he reads, and many a time has the story of the decadence of the Roman Empire been used to show that the vice and effeminacy which so often accompany high civilisations provide an automatic check to their extension, leaving the cultured races, as in the case of Rome, at the mercy of Goths and Huns. Nowadays it would seem as if the triumph of the savage is to be secured in other ways. No longer do we wait for outer barbarians to descend upon us. We merely arrange that the more brainy portion of the population shall retire from the task of peopling the world—a simple check to the overgrowth of hereditary genius. So at least it would appear from the facts detailed in a paper read at the annual meeting of the American Medical Association by Dr. Engelman in regard to the increasing sterility of American women. France has long bewailed the unwillingness of her daughters to become mothers of soldiers; lately England, the champion mother of nations, has also discovered that her fecundity is diminishing; and now it is the American woman, that final product of an exalted civilisation, who declines the common task and hands over to a crowd of immigrants from many nations and of all degrees the surely noble duty of peopling the American continent. Years ago, on the publication of a once famous (or infamous) pamphlet entitled the "Fruits of Philosophy," there was a great outcry. The protest was chiefly based upon moral grounds; but we are now beginning to find out how serious a canker immorality of the kind therein dealt with may prove to a whole nation. We are told that the fecundity of the American women was five children per family in the eighteenth century, 4·5 in the beginning of the nineteenth century, and that at the end of that century was between 1·8 and 2·1 per family, as against more than twice that figure among the immigrant population. Thus even in his own land the American is constantly being displaced by a mixture of American-born Irish and Germans, Italians, Scandinavians, Russians and Poles, beside the constant stream of immigrants. We do not say a word against these people. They are no doubt good, lusty Goths and Huns. But if there is anything to be proud of in being an American, a scion of that wonderful race (mixture if you like, but still practically by this time a race) by whom America has been made, it surely is matter for regret that at the present day the sterility of the American woman is greater and her fecundity is less than that of the women of any other nation except France. So much for the laws of evolution. This is the modern way in which a great people which has done its work tends to become ousted by more sexually vigorous races. The tree of knowledge is indeed full of danger to those who pick too freely of its fruit.

### The Stockport Isolation Hospital.

The angry feelings which have been excited at Stockport by the accusation brought against the management of the Stockport Isolation Hospital, do not seem to have as yet subsided, and at a recent meeting of the town council a letter was read from a solicitor, acting for the late matron, asking for a full and public inquiry into the whole matter. Without expressing any opinion on the merits of this case, we think that there is a moral which may usefully be drawn from the statements which have been published regarding the affair, namely, that when charges are brought against a public hospital it is best to hold a public inquiry at once. With the more rigid enforcement of the law in regard to the removal of infectious cases to hospital, the good management of isolation hospitals becomes a matter of personal interest to the head of every family to a degree which never used to be the case in times gone by, and the managers of such a hospital may be quite sure that when a "scandal" arises they cannot settle it by merely putting right what has been wrong. They must somehow or other convince the public that they are determined that things shall not be allowed to go wrong again, and that cannot be done except by removing every suspicion of hole and corner work. Nothing so quickly rouses public suspicion as any show of disinclination to public investigation. And after all the electors have a right to know.

